# Real-World Adherence and Effectiveness of Remote Patient Monitoring Among Medicaid Patients With Diabetes: Retrospective Cohort Study

**DOI:** 10.2196/45033

**Published:** 2023-08-22

**Authors:** Sulki Park, Hye-Chung Kum, Qi Zheng, Mark A Lawley

**Affiliations:** 1 Population Informatics Lab Texas A&M University College Station, TX United States; 2 Department of Industrial and Systems Engineering Texas A&M University College Station, TX United States; 3 Department of Health Policy and Management Texas A&M University College Station, TX United States; 4 Department of Epidemiology and Biostatistics Texas A&M University College Station, TX United States

**Keywords:** remote patient monitoring, telemonitoring, SMBG, telemedicine, Medicaid, blood glucose, diabetes, effectiveness, chronic condition, transmission rate, retrospective study

## Abstract

**Background:**

The prevalence of diabetes in the United States is high and increasing, and it is also the most expensive chronic condition in the United States. Self-monitoring of blood glucose or continuous glucose monitoring are potential solutions, but there are barriers to their use. Remote patient monitoring (RPM) with appropriate support has the potential to provide solutions.

**Objective:**

We aim to investigate the adherence of Medicaid patients with diabetes to daily RPM protocols, the relationship between adherence and changes in blood glucose levels, and the impact of daily testing time on blood glucose changes.

**Methods:**

This retrospective cohort study analyzed real-world data from an RPM company that provides services to Texas Medicaid patients with diabetes. Overall, 180 days of blood glucose data from an RPM company were collected to assess transmission rates and blood glucose changes, after the first 30 days of data were excluded due to startup effects. Patients were separated into adherent and nonadherent cohorts, where adherent patients transmitted data on at least 120 of the 150 days. *z* tests and *t* tests were performed to compare transmission rates and blood glucose changes between 2 cohorts. In addition, we analyzed blood glucose changes based on their testing time—between 1 AM and 10 AM, 10 AM and 6 PM, and 6 PM and 1 AM.

**Results:**

Mean patient age was 70.5 (SD 11.8) years, with 66.8% (n=255) of them being female, 91.9% (n=351) urban, and 89% (n=340) from south Texas (n=382). The adherent cohort (n=186, 48.7%) had a mean transmission rate of 82.8% before the adherence call and 91.1% after. The nonadherent cohort (n=196, 51.3%) had a mean transmission rate of 45.9% before and 60.2% after. The mean blood glucose levels of the adherent cohort decreased by an average of 9 mg/dL (*P*=.002) over 5 months. We also found that variability of blood glucose level of the adherent cohort improved 3 mg/dL (*P*=.03) over the 5-month period. Both cohorts had the majority of their transmissions between 1 AM and 10 AM, with 70.5% and 53.2% for the adherent and nonadherent cohorts, respectively. The adherent cohort had decreasing mean blood glucose levels over 5 months, with the largest decrease during the 6 PM to 1 AM time period (30.9 mg/dL). Variability of blood glucose improved only for those tested from 10 AM to 6 PM, with improvements of 6.9 mg/dL (*P*=.02). Those in the nonadherent cohort did not report significant changes.

**Conclusions:**

RPM can help manage diabetes in Medicaid clients by improving adherence rates and glycemic control. Adherence calls helped improve adherence rates, but some patients still faced challenges in transmitting blood glucose levels. Nonetheless, RPM has the potential to reduce the risk of adverse outcomes associated with diabetes.

## Introduction

The Center for Disease Control and Prevention [1] estimates that 11.3% of adults (over 37 million) in the United States have diabetes, with prevalence increasing to 23% for those 65 years or older. Further, the Center for Disease Control and Prevention [1] estimates that over 8 million adults with diabetes are undiagnosed, and that as many as 96 million adults have prediabetes. The number of adults with diabetes is expected to reach 60 million by 2060 [2].

Diabetes is the most expensive chronic condition in the United States totaling US $327 billion each year [3,4]. US $1 out of every US $4 in US health care costs is spent on caring for people with diabetes, and 61% of diabetes costs are spent for people aged 65 years or older. As the burden of diabetes grows, current systems of care will not keep pace with increasing demand, and, thus, developing more effective models and methods of care delivery is essential to the future diabetes care [5]. Emerging technologies, particularly those involving telehealth models are expected to play a major role, as will self-monitoring of blood glucose (SMBG) [6].

The benefits of SMBG for patients with type 1 diabetes mellitus and for insulin-treated patients with type 2 diabetes mellitus are well established, whereas those for non–insulin-treated patients have been questioned [7]. However, recent research addressing structured SMBG that includes education, SMBG profile, and feedback, indicates improved blood glucose levels in all persons with type 2 diabetes including those not treated with insulin [8]. Meanwhile, Schnell et al [6] reported several barriers to SMBG, including adherence, poor education, interpretation of readings, and appropriate actions.

Remote patient monitoring (RPM) with appropriate support has potential to give the solutions. RPM for patients with diabetes is a burgeoning area of research and practice, with the goal of deploying enabling technology to improve communication, monitoring and successful enactment of the plan of care [8]. Multiple systematic reviews and clinical trials found reductions in hemoglobin A_1c_ (HbA_1c_) values after using home blood glucose monitoring [8-14]. However, very few trials reported improvements on their fasting blood glucose [15-17], which is a more accurate predictor for diagnosis of diabetes [18].

Continuous glucose monitoring (CGM) is also emerging as a useful tool for real-time monitoring of blood glucose in clinical and public diabetes management settings, and for assessing the impact of treatment and lifestyle on daily changes in blood glucose levels [19]. However, several potential common patient-reported barriers of CGM use include sensor insertion with pain or problems of high costs, accidental removal of the device or the adhesive strip, and skin reactions of sensor adhesion. Older people living in poverty could be reluctant to use CGM, and studies show that it is more expensive than SMBG [20]. For those patients, well-structured SMBG could be the best option.

The aims of this retrospective cohort study using data from an RPM company serving Texas Medicaid patients are to (1) determine how well Texas Medicaid patients adhered to daily SMBG-based RPM protocols, (2) examine the relationship between adherence and changes in blood glucose levels associated with daily monitoring, and (3) investigate the impact of daily testing time on the mean and variance of SMBG readings over the study period.

## Methods

### Data Source

We conducted a retrospective cohort study using SMBG data obtained from an RPM company that provides remote monitoring services to Texas Medicaid patients. We obtained data covering the period 2016 to 2018 for Medicaid clients with diabetes or hypertension (see [21] for our analysis for patients with hypertension). The data for patients with diabetes includes information about patient demographics, primary doctor, blood glucose transmission date and values, alert time, and notes from clinical calls.

### RPM Protocol

Monitoring services were provided following physician prescription and Texas Medicaid approval. Once approval was granted, a company technology deployer visited the patient’s home to set up the necessary equipment, which included a Food and Drug Administration–approved SMBG device with Bluetooth capabilities (TD-3223 [TaiDoc Technology] or D40d [ForaCare]. The deployer also provided training to the patients, which followed American Medical Association guidelines [22]. Training included education on how to use the equipment to take proper readings and information about the company’s protocols for responding to patients’ technical or clinical needs. They also asked patients to select a daily time by which they would test and transmit their blood glucose levels. If transmission did not occur by that time, an automated alert prompted a company staff member to make a reminder call (referred to as an adherence call) to the patient to troubleshoot any technical issues and to remind the patient to test and transmit the blood glucose levels. If the levels fell outside the physician-defined acceptable ranges, an automated clinical alert was transmitted to a company nurse. The nurse placed a clinical phone call to the patient, categorized the extent of concern following a protocol, and contacted the physician by email for the lowest level of concern and by both email and phone call for more severe concerns. The physician reviewed and signed off on weekly summary reports for the enrolled patients. Under Texas Medicaid rules, a request for reauthorization of the RPM service was made every 60 days when the physician prescribed additional monitoring. The detailed RPM protocol is described in our previous paper on hypertension [21].

### Sample

**Figure 1:**
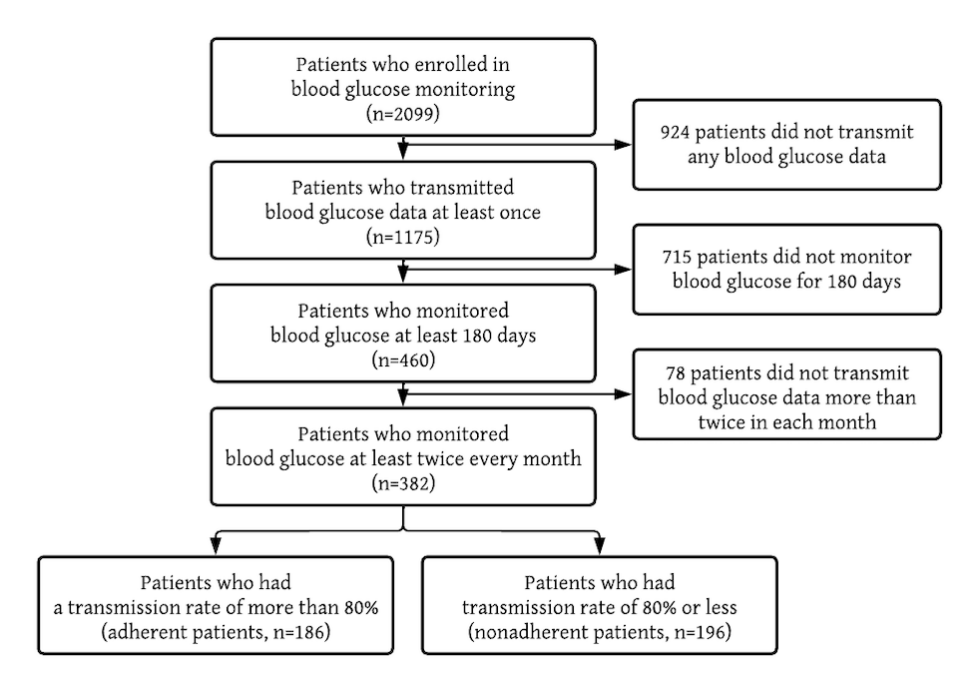
Patient enrollment flowchart.

Only clients with 180 days or more on the RPM service were included in this study to have sufficient follow-up time. The first 30 days were regarded as a startup period during which the patients learned to use the equipment to measure their glucose values, and were excluded from this study; thus, the study period was 150 days (months 1-5). If the blood glucose levels of the patient were transmitted only once or less in any month, that patient was excluded from this study. The patients were separated into adherent and nonadherent cohorts; adherent patients were those who tested blood glucose levels on at least 120 of the 150 days (at least 80% of the days). This is consistent with the past study on SMBG [23]. See Figure 1 for sample flow chart.

### Experimental Design

As our study focused on daily basis RPM, we selected the last blood glucose reading of the day in cases where a patient transmitted multiple readings due to technical issues or by choice. This decision was made to account for cases where a recheck was needed due to a misreading during the first attempt. Additionally, in situations where multiple readings were received simultaneously due to technical issues, selecting the last reading ensured that the most recent and up-to-date data was used for follow-up.

Transmission rates ([total number of patients who transmitted readings] / [total number of patients] × 100) before and after the adherence calls were recorded each day, as was the number of adherence calls made. In cases where multiple calls were made to a patient, we selected the first call time for our analysis. We included all attempted adherence calls, even those that the patients did not answer, because, in these cases, voice mail was left whenever possible.

Improvements in blood glucose control during the study period were studied using the mean and SD of blood glucose levels at month 1 and 5 for each patient. As glycemic variability is an important metric to consider when assessing glycemic control, changes in SD were analyzed [24]. We report out the group mean of these individual patient measures for the month.

In addition, to account for the natural fluctuations in blood glucose levels throughout the day, we also conduct subgroup analysis of blood glucose levels based on their testing time—between 1 AM and 10 AM, 10 AM and 6 PM, and 6 PM and 1 AM. The thresholds were based on the patient daily routine and the actual blood glucose levels presented in Multimedia Appendix 1. Afterward, to evaluate improvements in blood glucose control considering testing time, we compared the mean and SD of each patient's levels at months 1 and 5 within each testing time range. This analysis specifically focused on patients who consistently transmitted their data within the specified time intervals every month. Therefore, patients can be included in multiple time intervals depending on their testing habits.

### Statistical Analysis

We used chi-square tests for categorical variables and *t* tests for continuous variables to compare the patient baseline characteristics between population subgroups. We also performed *z* tests for the equality of the 2 proportions to compare the transmission rates between 2 subgroups. In addition, paired *t* tests were performed to analyze the blood glucose changes from month 1 to month 5 for each subgroup. Two-sample *t* tests were performed to compare the blood glucose changes between the subgroups. Analyses were conducted using SAS (version 9.4; SAS Institute).

### Ethics Approval

This secondary retrospective cohort study protocol and procedures have been reviewed and approved by the institutional review board (IRB) of Texas A&M University as an expedited study with an annual administrative check in (IRB2018-0166D). There was no risk to the participants’ health from participation in this study because data were collected either as part of patients’ routine care or for billing purposes and used for quality improvement. The IRB determined that the study presented minimal risk to participants given no interaction or intervention with patients. In addition, to further safeguard participant confidentiality we took several measures following industry standards. First, all study data were pseudonymized at data ingest and only the coded data with randomly generated IDs were used for analysis. Second, all data were stored and analyzed on a Health Insurance Portability and Accountability Act–compliant data enclave using industry standard encryption, routine updates, routine vulnerability scans, access controls, and training that ensure all data are secure at all times. These measures were implemented to ensure that the participants' rights and interests were upheld throughout the study, that any potential risks associated with the research were minimized, and research was conducted in a responsible and respectful manner, while still gathering valuable insights from the data.

## Results

### Patient Characteristics

A total of 2099 clients enrolled in RPM for blood glucose control (Figure 1). Of the 2099 patients, 460 (21.9%) enrolled 180 days or more, and 382 (18.2%) tested their blood glucose levels at least twice every month. Of the 382 patients, 186 (48.7%) were adherent and the other 196 (51.3%) were nonadherent to the RPM.

**Figure 2:**
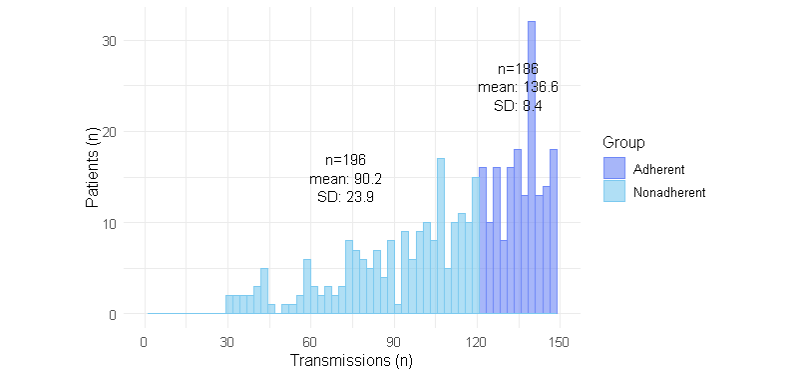
Number of transmissions per patient over 150 days for adherent and nonadherent cohorts (N=382). Adherent patients were those who tested blood glucose levels on at least 120 of the 150 days (at least 80% of the days) and nonadherent patients were those who tested less than 80% of the days.

Over the 150-day period, the 382 patients generated a total of 43,076 blood glucose transmissions, with 25,396 transmissions from the adherent cohort and 17,680 transmissions from the nonadherent cohort. On average, the adherent cohort sent 136.6 (SD 8.4) transmissions during the study period, which corresponded to an average of 27.3 (SD 3.5) readings per month (Figure 2). In contrast, the nonadherent cohort transmitted a mean of 90.2 (SD 23.9) transmissions, which corresponded to an average of 18 (SD 7.2) readings per month.

Table 1 shows the demographic information including age, gender, and area of residence. The mean age of the patients at starting the service was 70.5 (SD 11.8) years. More than half of the patients (255/382, 66.8%) were women, and predominantly from McAllen in south Texas (340/382, 82%), and urban areas (351/382, 91.9%).

The characteristics across the 2 cohorts were similar, although the adherent cohort had a lower proportion of women (*P*=.01), with more of them living in McAllen (*P*=.02).

**Figure 3:**
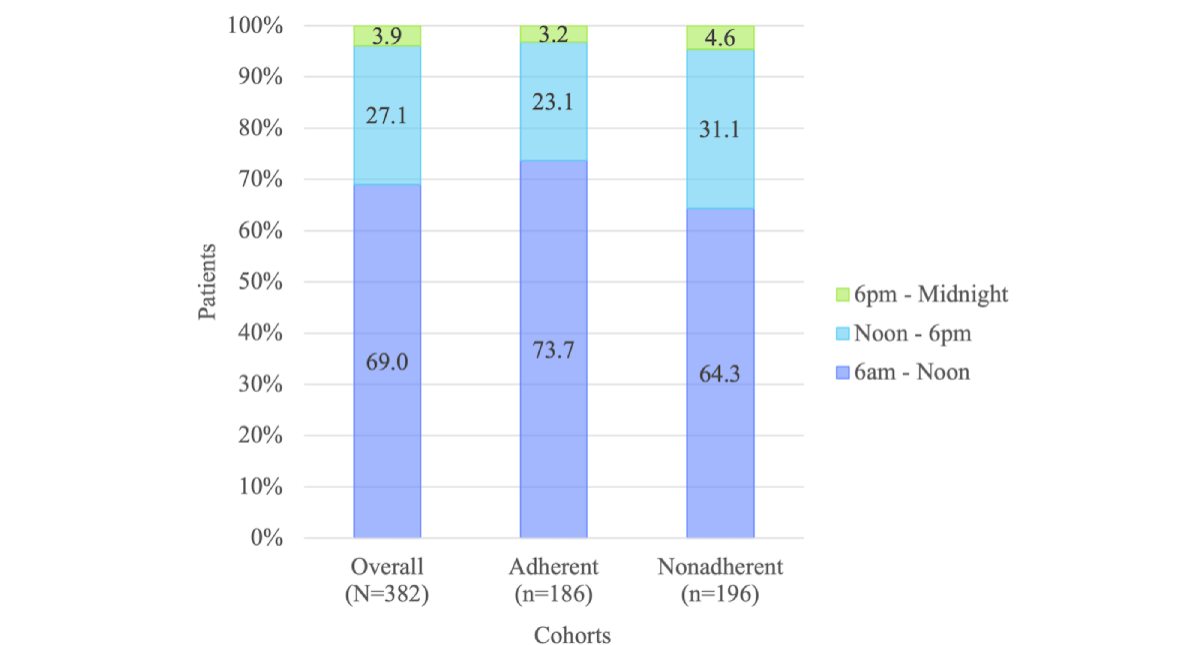
Assigned adherence alert time for overall, adherent, and nonadherent cohorts (N=382). Adherent patients were those who tested blood glucose levels on at least 120 of the 150 days (at least 80% of the days) and nonadherent patients were those who tested less than 80% of the days.

Figure 3 shows assigned adherence alert time. A majority of participants set their adherence alerts in the morning between 6 AM and noon (263/382, 69.0%), with 73.7% (137/186) and 65.6% (126/196) of the adherent and nonadherent cohorts preferring morning, respectively (*P*=.048).

**Table 1:** Demographics for overall, adherent, and nonadherent cohorts (N=382).

Characteristics		Values				
		Overall (N=382)	Adherent (n=186)	Nonadherent (n=196)	*P* value	
Age (years), mean (SD)		70.5 (11.8)	69.7 (10.9)	71.3 (12.6)	.20	
Women, n (%)		255 (66.8)	112 (60.2)	143 (73)	.01	
**Area of residence, n (%)**						.02
	Dallas	23 (6)	7 (3.8)	16 (8.2)		
	Houston or San Antonio	19 (5)	5 (2.7)	14 (7.1)		
	McAllen	340 (89)	174 (93.6)	166 (84.7)		
**Urban-rural classification, n (%)**						.48
	Urban	351 (91.9)	169 (90.9)	182 (92.9)		
	Suburban or rural	31 (8.1)	17 (9.1)	14 (7.1)		
Transmissions, n		43,076	25,396	17,680	N/A^a^	

^a^N/A: not applicable.

### Daily Adherence

**Figure 4:**
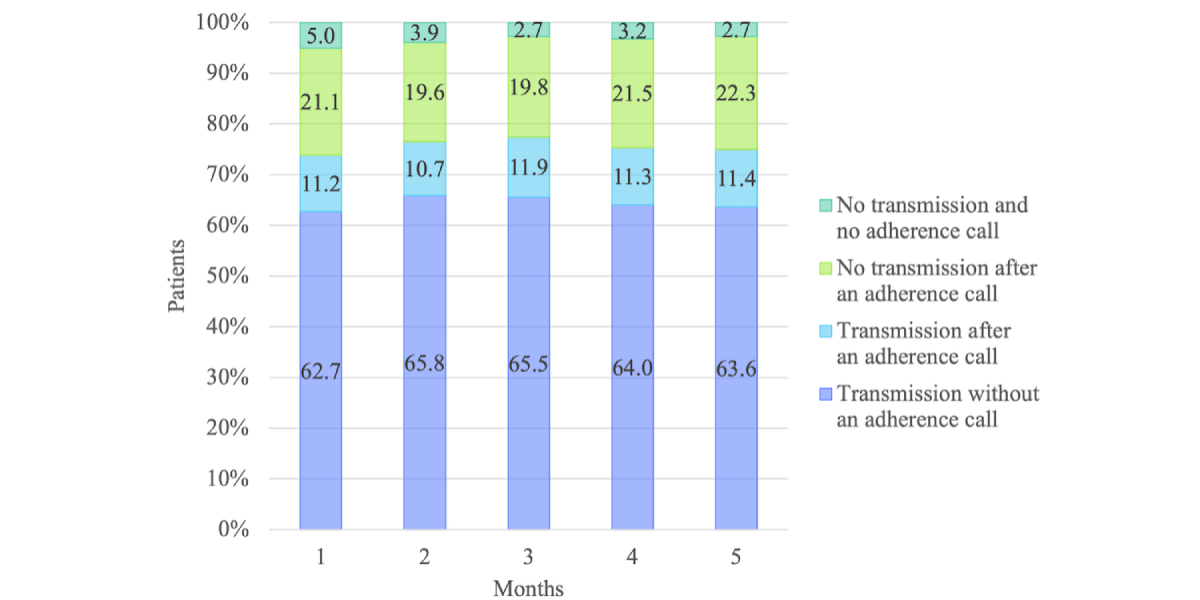
Average daily transmission rates by month for all patients over 150 days of remote monitoring (N=382). Adherent patients were those who tested blood glucose levels on at least 120 of the 150 days (at least 80% of the days) and nonadherent patients were those who tested less than 80% of the days. The transmission rates (= [total number of patients who transmitted readings] / [total number of patients] × 100) were calculated daily, then we calculated the average of these daily measurements for each month.

Figure 4 shows the transmission rate over the 5-month (150-day) period. The overall mean transmission rates across all 5 months were 64.3% before the adherence call and 75.6% afterward. The mean transmission rates for the first month were 62.7% and 73.8% before and after the call, respectively. The transmission rates before the adherence call declined from 65.8% in the second month to 63.6% in the fifth month, while those rates after the call reached the highest of 77.4% in the third month and then declined to 75% in the fifth month. As indicated in the second block of each bar in Figure 4, an average of 11.3% of the data transmissions were received after an adherence call. However, an average of 20.9% of the participants did not transmit the data after an adherence call.

**Figure 5:**
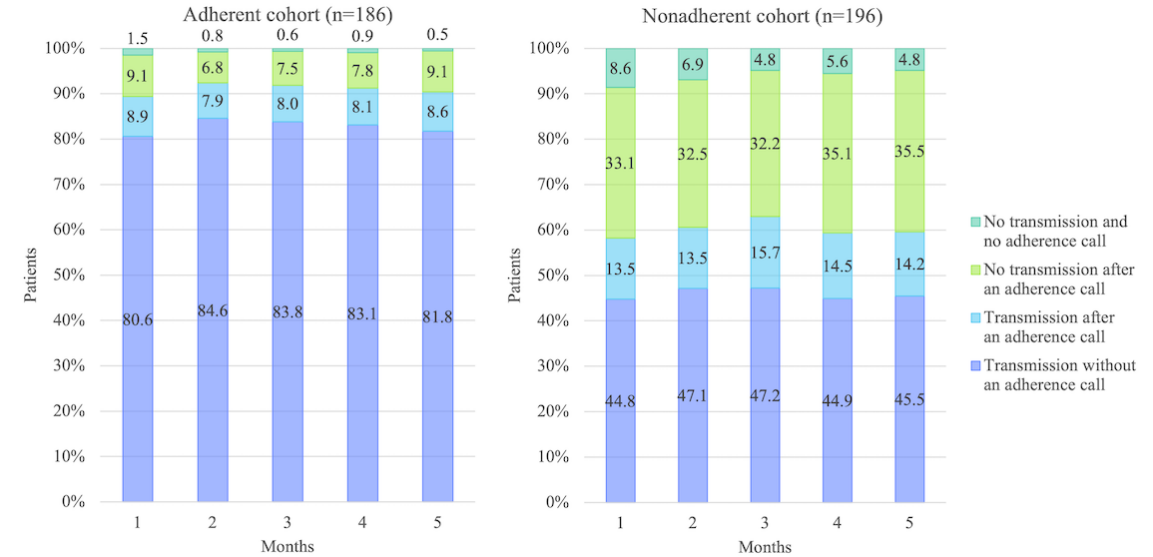
Average daily transmission rates by month for the adherent and nonadherent cohort over 150 days of remote monitoring (N=382). Adherent patients were those who tested blood glucose levels on at least 120 of the 150 days (at least 80% of the days) and nonadherent patients were those who tested less than 80% of the days. The transmission rates (= [total number of patients who transmitted readings] / [total number of patients] × 100) were calculated daily, then we calculated the average of these daily measurements for each month.

The adherent and nonadherent cohorts showed large difference in those rates (Figure 5). The adherent cohort was much more likely to transmit data without an adherence call, with an overall mean transmission rate of 82.8% compared with only 45.9% for the nonadherent cohort (*P*<.001). After the adherence reminder call, these values increased to 91.1% and 60.2% (*P*<.001), respectively.

The mean transmission rates for the first month were 80.6% before the adherence call and 89.4% after the call for the adherent cohort. These values reached the highest of 84.6% and 92.4% in the second month and then declined to 81.8% and 90.4% before and after the call, respectively, in the fifth month. On the other hand, the mean transmission rates for the first month were 44.8% and 58.2% before and after the call for the nonadherent cohort. These values reached the highest of 47.2% and 63% in the third month and declined to 45.5% and 59.6% before and after the call, respectively, until the fifth month. On average, an additional 8.3% of the transmissions were received after an adherence reminder call from the adherent cohort, while an additional 14.3% transmissions were received after the call from the nonadherent cohorts (*P*=.07).

The percentage of participants not transmitting after an adherence reminder call was, on average, 8.1% for the adherent cohort and 33.7% for the nonadherent cohort (*P*<.001). We noted that, on average, 6.2% of the nonadherent participants who did not transmit data by the specified time failed to receive an adherence call. This value decreased to 4.8% in the fifth month of monitoring. In contrast, only 0.9% of the adherent cohort who did not transmit data failed to receive an adherence reminder call.

### Adherence Reminder Call

Overall, the adherent and nonadherent cohorts received 4616 (24.3%) and 14,401 (75.7%) adherence reminder calls over the 5 months. On average, 1 patient in the adherent cohort received 25.4 adherence calls (SD 19.6) over 5 months, and that was 6.1 (SD 5.2) calls per month. On the other hand, one in the nonadherent cohort received 74.2 (SD 34) adherence calls over 5 months, and that was 15.4 (SD 8.4) calls per month.

Of those 4616 adherence calls to the adherent cohort, 50.7% (n=2341) helped a patient to transmit data, while of those 14,401 adherence calls to the nonadherent cohort, only 29.5% (n=4247) resulted in a patient transmitting data.

A company staff member called a patient within 30 minutes after an adherence alert at median (26 and 27 minutes for the adherent and nonadherent cohorts, respectively). And a patient in the adherent cohort transmitted data in 64.5 minutes after the adherence reminder call, while it took 95 minutes for the nonadherent cohort at median.

### Relationship Between Adherence to RPM and Changes in Blood Glucose Control

Overall, we found that mean blood glucose levels of the adherent cohort decreased by an average of 9 mg/dL (*P*=.002) over 5 months which dropped from 147.2 (SD 48.1) mg/dL at month 1 to 138.2 (SD 30.3) mg/dL at month 5 (Table 2).

We also found that variability of blood glucose level of the adherent cohort improved 3 mg/dL (*P*=.03) over the 5-month period. However, mean and variability of blood glucose levels of the nonadherent cohort did not significantly change over time.

**Table 2:** Blood glucose changes between months 1 and 5 (N=382).

Month		Adherent cohort (n=186)	Nonadherent cohort (n=196)	*P* value^a^
**Month 1**				
	Transmissions, n	4990	3421	N/A^b^
	Monthly mean (mg/dL), mean (SD)	147.2 (48.1)	154.9 (50.2)	.13
	Monthly variability (mg/dL), mean (SD)^c^	33.3 (24.5)	40 (25.1)	.01
**Month 5**				
	Transmissions, n	5040	3505	N/A
	Monthly mean (mg/dL), mean (SD)	138.2 (39.6)	157.1 (48.7)	<.001
	Monthly variability (mg/dL), mean (SD)^c^	30.3 (19.3)	39.1 (22.9)	<.001
**Comparison of monthly mean between months 1 and 5**				
	Values, mean (SD)	–9 (38.7)	2.2 (37)	.004
	*P* value^d^	.002	.41	N/A
**Comparison of monthly variability between months 1 and 5**				
	Values, mean (SD)	–3 (18.6)	–0.9 (18.9)	.27
	*P* value^d^	.03	.49	N/A

^a^A 2-tailed independent *t* test was performed to compare the blood glucose changes between the adherent and nonadherent cohorts.

^b^N/A: not applicable.

^c^SD was calculated to address monthly glucose variability.

^d^A 2-tailed paired *t* test was performed to analyze the differences in blood glucose between months 1 and 5 for each cohort.

### Blood Glucose Testing Time and Level Differences

To investigate the impact of testing time on blood glucose improvements, we conducted an analysis by grouping blood glucose levels into 3 categories—between 1 AM and 10 AM, 10 AM and 6 PM, and 6 PM and 1 AM. These time thresholds were determined based on patients' daily routines and the actual blood glucose levels presented in Multimedia Appendix 1.

Among the adherent cohort, the majority of transmissions occurred between 1 AM and 10 AM, with 69.3% (n=3459) in month 1 and 72.2% (n=3638) in month 5 (Table 3). Additionally, 18.7% (n=935) and 17.1% (n=862) took place between 10 AM and 6 PM, while 11.9% (n=596) and 10.7% (n=540) occurred between 6 PM and 1 AM for month 1 and month 5, respectively.

To assess improvements in blood glucose control with regard to testing time, the mean and SD for each patient at month 1 and month 5 for each testing time range were calculated. We found that over the 5-month period, the mean blood glucose levels for the adherent cohort tested from 1 AM to 10 AM, 10 AM to 6 PM, and 6 PM to 1 AM decreased by an average of 6.5 mg/dL (*P*=.09), 12.1 mg/dL (*P*=.02), and 30.9 mg/dL (*P*=.004), respectively.

Furthermore, we noted a significant improvement in the variability of blood glucose levels among those who tested between 10 AM and 6 PM, with a decrease of 6.9 mg/dL (*P*=.02) over the 5-month period.

Regarding the nonadherent cohort, a similar trend was observed, with the majority of transmissions occurring between 1 AM and 10 AM. Specifically, there were 1784 (52.1%) transmissions in month 1 and 1878 (53.6%) transmissions in month 5 (Table 4). Furthermore, there were 35.2% (n=1203) and 35.3% (n=1236) of transmissions between 10 AM and 6 PM, and 12.7% (n=434) and 11.2% (n=391) between 6 PM and 1 AM, in month 1 and month 5, respectively.

However, we did not observe any significant changes in the mean and variability of blood glucose levels among the nonadherent cohort.

**Table 3:** Blood glucose changes from month 1 to 5 in each time interval for the adherent cohort (N=186).

Month			Transmissions between 1:00 AM and 9:59 AM (n=150)^a^		Transmissions between 10:00 AM and 5:59 PM (n=56)^a^		Transmissions between 6:00 PM and 12:59 AM (n=28)^a^
**Month 1**							
	Transmissions, n	3459		935		596	
	Monthly mean (mg/dL), mean (SD)	136.9 (49.1)		164.9 (51.6)		205.6 (59.2)	
	Monthly variability (mg/dL), mean (SD)^b^	24 (16.2)		41.5 (26.8)		53.8 (25.2)	
**Month 5**							
	Transmissions, n	3638		862		540	
	Monthly mean (mg/dL), mean (SD)	130.4 (37.7)		152.9 (46.5)		174.6 (44.8)	
	Monthly variability (mg/dL), mean (SD)^b^	24.1 (16.4)		34.5 (22)		50 (22)	
**Comparison of monthly mean between months 1 and 5**							
	Values, mean (SD)	–6.5 (46.1)		–12.1 (38.8)		–30.9 (52.1)	
	*P* value^c^	.09		.02		.004	
**Comparison of monthly variability between months 1 and 5**							
	Values, mean (SD)	0.04 (15.1)		–6.9 (21.8)		–3.8 (22.1)	
	*P* value^c^	.97		.02		.37	

^a^Every patient who transmitted in specified time period every month was selected. Therefore, patients in each time interval are not mutually exclusive.

^b^SD was calculated to address monthly glucose variability.

^c^A 2-tailed paired *t* test was performed to analyze the differences in blood glucose between months 1 and 5 for each cohort.

**Table 4:** Blood glucose changes between month 1 and month 5 in each time interval for the nonadherent cohort (N=196).

Month			Transmissions between 1:00 AM and 9:59 AM (n=115)^a^		Transmissions between 10:00 AM and 5:59 PM (n=94)^a^		Transmissions between 6:00 PM and 12:59 AM (n=26)^a^
**Month 1**							
	Transmissions, n	1784		1203		434	
	Monthly mean (mg/dL), mean (SD)	140.1 (40.1)		169.5 (55.2)		190.6 (69.5)	
	Monthly variability (mg/dL), mean (SD)^b^	27.9 (18)		45.5 (29.6)		56.1 (23.1)	
**Month 5**							
	Transmissions, n	1878		1236		391	
	Monthly mean (mg/dL), mean (SD)	145.9 (40)		162.4 (50.2)		191.7 (62.2)	
	Monthly variability (mg/dL), mean (SD)^b^	28.1 (17.3)		40.6 (22.5)		59.5 (25)	
**Comparison of monthly mean between months 1 and 5**							
	Values, mean (SD)	5.7 (35.7)		–7.0 (39.7)		1.1 (51.7)	
	*P* value^c^	.09		.09		.91	
**Comparison of monthly variability between months 1 and 5**							
	Values, mean (SD)	0.2 (16.7)		–5 (28.4)		3.4 (20.9)	
	*P* value^c^	.88		.09		.41	

^a^Every patient who transmitted in specified time interval every month were selected. Therefore, patients in each time interval are not mutually exclusive.

^b^SD was calculated to address monthly glucose variability.

^c^A 2-tailed paired *t* test was performed to analyze the differences in blood glucose between months 1 and 5 for each cohort.

## Discussion

### Principal Results

Achieving target glycemic control is crucial in managing diabetes and preventing complications, which can significantly impact a patient's quality of life [25]. Diabetes management requires regular monitoring of blood glucose levels and frequent communication with health care professionals to adjust treatment plans [16]. However, accessibility to diabetes specialists may be limited in some areas, and this can affect the quality of care that patients receive. Therefore, alternative RPM technology has gained increasing attention to alleviate this burden.

Recent randomized clinical trials have shown promising results for RPM interventions in improving glycemic control [15-17]. Boaz et al [15] found that patients with RPM experienced a 15 mg/dL decrease in fasting blood glucose levels while patients without RPM experienced an increase over a 6-month period. Similarly, Jeong et al [16] reported 7.6 mg/dL and 12.3 mg/dL improvements in blood glucose levels for patients with telemonitoring (SMBG + automated message support) and telemedicine (SMBG + video communication) within 24 weeks, respectively, compared to those with conventional monitoring without blood glucose transmission. Franc et al [17] found that twice as many patients in the RPM group achieved target fast blood glucose compared to the control group receiving standard care. These findings from randomized clinical trials suggest that RPM has the potential to enhance patient care and improve outcomes in diabetes management. However, the effectiveness of RPM in the real-world setting is still unclear, with limited data available.

The objective of this retrospective cohort study was to assess the effectiveness of SMBG-based RPM among Medicaid clients with diabetes in a real-world setting. Specifically, the study aimed to evaluate the clients' adherence to RPM and investigate any changes in their blood glucose levels during the 5-month RPM period. The RPM service provided patients with diabetes with adherence support to monitor their glucose levels daily and receive immediate clinical feedback. The findings of this study indicate that the overall adherence rate for the RPM system among Medicaid clients was over 70% with the help of adherence calls. Moreover, nearly half of the clients (adherent cohort, 186/382, 48.7%) achieved remarkably high adherence levels of approximately 90%, which were sustained throughout the study period with the help of adherence calls.

The study findings reveal that adherence calls played a significant role in improving clients' adherence to blood glucose monitoring, resulting in a 10% increase in adherence rates throughout the 5-month RPM period. Notably, more than half of the clients (nonadherent cohort, 196/382, 51.3%) showed an impressive 14% improvement in adherence due to the calls, while the other clients (adherent cohort) showed an 8% improvement. Despite a slight decline in adherence over time, the adherence calls helped to maintain adherence levels.

However, the study also revealed that the adherence calls faced some challenges, with approximately 80 of the 382 (20.9%) patients failing to transmit their blood glucose levels each day, despite the reminders. The nonadherent cohort was particularly impacted, with approximately 66 out of the 196 (33.7%) patients failing to transmit their blood glucose levels daily. In contrast, only 15 out of the 186 (8.1%) patients in the adherent cohort did not transmit their blood glucose levels daily. Despite these challenges, the study demonstrated the overall effectiveness of adherence calls in supporting both groups.

During the study period, the adherent cohort showed a decrease in mean blood glucose values, indicating improved glycemic control. Additionally, the glycemic variability, as measured by the SD of blood glucose values, decreased only for the adherent cohort. This finding is particularly important because glycemic variability has been shown to be closely associated with the risk of adverse clinical outcomes and complications [24].

Our study found that blood glucose levels varied considerably throughout the day, with values being lowest in the morning and gradually increasing as the day progressed. Notably, we found that blood glucose values increased from under 120 mg/dL in the early morning to over 180 mg/dL at night. Interestingly, our findings revealed that the adherent cohort experienced significant improvements in blood glucose levels during the afternoon and night when values are typically higher and more variable. However, we did not observe any significant changes in the nonadherent cohort.

This program collected blood glucose readings from 382 patients which accounted for 43,076 days of readings in total. Of these, the majority (36,069, 84%) had a single reading in a day, while 13% (5439) had 2 readings, and 3.6% (1568) had 3 or more readings. Although the program was designed to send a reading once a day, some patients used the RPM device to check their blood glucose levels more than once or were asked to retake the reading during a clinical call to address potential issues such as misreading values or if the patient seemed confused when taking readings. The company provided clinical support regardless of how many times patients sent readings outside the predefined ranges in a day. When multiple readings were taken on the same day, about 30% (n=2067) were taken within the same time range, while 60.3% (n=4223) had the first reading taken between 1 AM and 10 AM, with subsequent readings taken at other times.

For days with all readings taken in the same time range, the median difference between the first and last reading was 16 mg/dL. However, for those with readings taken between 1 AM and 10 AM and at other times, the median difference was 49 mg/dL. This difference appears reasonable given that the mean blood glucose level was 135 mg/dL between 1 AM and 10 AM and 186.1 mg/dL between 6 PM and 1 AM. For days with multiple readings, we selected the latest reading for our analysis. This was done to account for cases where a recheck was needed due to a misreading during the first attempt and cases where multiple readings were received simultaneously due to technical issues. Additionally, the last reading is the most recent situation in which the patient would be supported by the program. Furthermore, by analyzing the readings according to their testing time, we were able to minimize the potential issues that may arise from variations in the testing time. This approach allowed us to focus on the blood glucose levels themselves, rather than being influenced by the timing of the tests.

Importantly, our study showed that a single daily transmission of SMBG data was associated with positive improvements in blood glucose levels, despite the considerable variation throughout the day. This technology may be enabling several interacting factors that contribute to these improvements, such as more timely interaction with providers, improved provider awareness, patient adherence to monitoring protocols, reminders, and clinical calls to assist patients when issues arise and to encourage healthy behaviors through a mechanism to better engage with their health, and so forth. All these factors may work together to encourage and empower patients in improving their overall self-management. Fortunately, individuals with diabetes have shown good acceptance of technology [26], which suggests that this model of care has potential. However, improving self-efficacy and adherence among those who are less inclined to participate consistently remains a significant challenge.

### Limitations

This study has several limitations that should be noted. First, we had no information on patient medications, activity levels, carbohydrate consumption, or other factors that may have influenced glucose levels. Additionally, we were unable to obtain HbA_1c_ data or determine the type of diabetes (type 1 or type 2) that the patients had. Furthermore, since the study only included patients who were referred to the monitoring program by their physician, we were unable to include a control group for comparison. Finally, the monitoring protocol required data to be transmitted once per day, which is less frequent than the general home blood glucose monitoring guidelines recommend [27].

### Conclusions

This study highlights the potential benefits of RPM for diabetes management among Medicaid clients. The study found that the overall adherence rate for the RPM system was over 70%, with approximately half of the clients achieving a remarkably high adherence rate of approximately 90%. Adherence calls played a significant role in improving clients' adherence to blood glucose monitoring, resulting in a more than 10% increase in adherence rates throughout the 5-month RPM period for all patients. The adherent cohort showed a decrease in mean blood glucose values and a decrease in glycemic variability, indicating improved glycemic control. However, challenges were faced with approximately 20% of patients failing to transmit their blood glucose levels daily, even with adherence calls. Nonetheless, this study suggests that RPM can be an effective tool to enhance diabetes management among Medicaid clients, with the potential to reduce the risk of adverse clinical outcomes and complications associated with diabetes.

## Appendix

Multimedia Appendix 1 Mean blood glucose levels and proportion of transmissions in each hour for the adherent and nonadherent cohort (N=382).

## Abbreviations

CGM: continuous glucose monitoring
HbA_1c_: hemoglobin A_1c_
IRB: institutional review board
RPM: remote patient monitoring
SMBG: self-monitoring of blood glucose

## Data Availability

The data sets analyzed during this study are not publicly available due to the inclusion of patients' personal information but may be available from the corresponding author on reasonable request and appropriate approvals.
