# Supplementary material for: Real-World Adherence and Effectiveness of Remote Patient Monitoring Among Medicaid Patients With Diabetes: Retrospective Cohort Study
Source: J Med Internet Res. 2023 Aug 22;25:e45033. doi: 10.2196/45033 (PMC10481216; doi:10.2196/45033)
Supplement: Multimedia Appendix 1 [file jmir_v25i1e45033_app1.docx]

**Mean blood glucose levels and proportion of transmissions in each hour for the adherent and nonadherent cohort (N=382).**


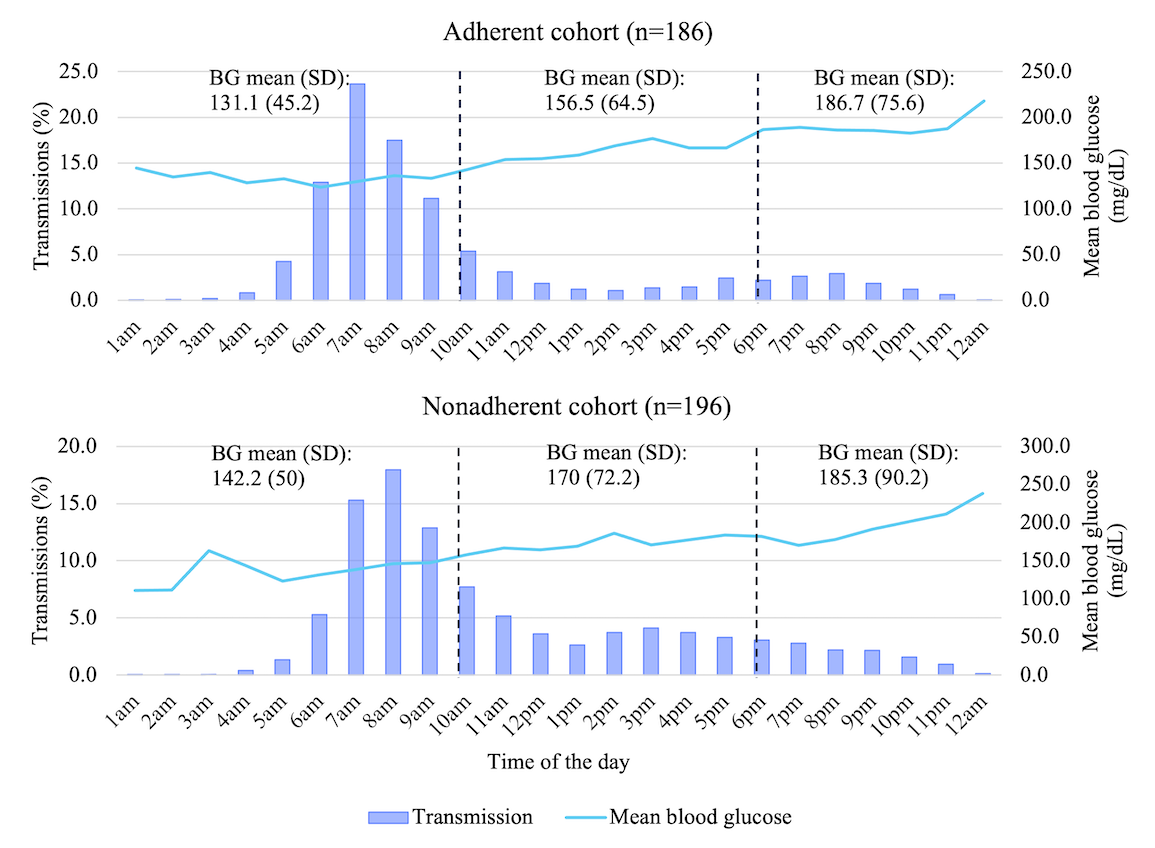


**NOTE** Adherent patients were those who tested blood glucose levels on at least 120 of the 150 days (at least 80% of the days) and nonadherent patients were those who tested less than 80% of the days. The proportion of transmissions and mean blood glucose levels were calculated using every reading sent during that time period.

**Grouping blood glucose levels based on testing time**

Blood glucose levels generally exhibit the lowest values in the morning before the first meal, and they fluctuate throughout the day, as illustrated in the graph above. Considering the possibility that the observed improvements in blood glucose levels among the adherent cohort in our manuscript might be associated with the testing time, we performed a subgroup analysis by categorizing transmissions according to their testing time and assessed the changes.

Given that blood glucose levels typically rise after meals, we decided to group the readings as follows: before the first meal (breakfast/lunch), after the first meal, and after dinner (i.e., 1 AM to 10 AM, 10 AM to 6 PM, and 6 PM to 1 AM). Notably, we observed significant increases in blood glucose levels between 9 AM and 10 AM, from 133.5 mg/dL to 143.1 mg/dL, and between 5 PM and 6 PM, from 166.4 mg/dL to 186.6 mg/dL, among the adherent cohort.

This is a Multimedia Appendix to a full manuscript published in the J Med Internet Res. For full copyright and citation information see http://dx.doi.org/10.2196/jmir.45033.
